# Oral and cardiometabolic health through the lens of biobanks and large-scale epidemiologic research

**DOI:** 10.3389/froh.2026.1774868

**Published:** 2026-03-13

**Authors:** Nada Tawfig Hashim, Rasha Babiker, Vivek Padmanabhan, Md Sofiqul Islam, Sivan Padma Priya, Nallan C. S. K. Chaitanya, Riham Mohammed, Shahistha Parveen Dasnadi, Ayman Ahmed, Bakri Gobara Gismalla, Muhammed Mustahsen Rahman

**Affiliations:** 1 Department of Periodontics, RAK College of Dental Sciences, RAK Medical & Health Sciences University, Ras-AlKhaimah, United Arab Emirates; 2 Department of Physiology, RAK College of Medical Sciences, RAK Medical & Health Science University, Ras-AlKhaimah, United Arab Emirates; 3 Department of Pediatric and Preventive Dentistry, RAK College of Dental Sciences, RAK Medical & Health Sciences University, Ras-AlKhaimah, United Arab Emirates; 4 Department of Operative Dentistry, RAK College of Dental Sciences, RAK Medical and Health Sciences University, Ras-AlKhaimah, United Arab Emirates; 5 Department of Oral Pathology, RAK College of Dental Sciences, RAK Medical and Health Sciences University, Ras-AlKhaimah, United Arab Emirates; 6 Department of Oral Medicine and Radiology, RAK College of Dental Sciences, RAK Medical & Health Sciences University, Ras-AlKhaimah, United Arab Emirates; 7 Department of Oral Surgery, RAK College of Dental Sciences, RAK Medical & Health Sciences University, Ras-AlKhaimah, United Arab Emirates; 8 Department of Orthodontics, RAK College of Dental Sciences, RAK Medical & Health Sciences University, Ras Al Khaimah, United Arab Emirates; 9 Department of Periodontology and Implantology, Nile University, Khartoum, Sudan; 10 Department of Oral Rehabilitation, Faculty of Dentistry, University of Khartoum, Khartoum, Sudan

**Keywords:** biobanks, cardiometabolic disease, cardiovascular disease, inflammation, large-scale epidemiologic studies, microbiome, oral–systemic health

## Abstract

Oral diseases and cardiometabolic disorders are among the most prevalent non-communicable conditions worldwide and share common inflammatory, metabolic, and social determinants. Over the past two decades, growing evidence has linked poor oral health—particularly periodontitis and tooth loss—to cardiometabolic outcomes such as type 2 diabetes and cardiovascular disease. However, progress in this field has long been constrained by fragmented data systems and limited availability of large-scale resources capturing both oral health exposures and cardiometabolic endpoints. Recent advances in biobank infrastructure, population-based cohorts, and electronic health record linkage have transformed this landscape, enabling robust secondary analyses at unprecedented scale. This narrative review synthesizes current evidence from major biobanks and large-scale epidemiologic datasets relevant to oral–cardiometabolic research. We describe how oral health and cardiometabolic outcomes are operationalized across data ecosystems, critically appraise the strengths and limitations of key resources, and integrate epidemiologic findings with established biological mechanisms, including chronic systemic inflammation, microbial dysbiosis, metabolic dysfunction, and vascular impairment. We further highlight the bidirectional nature of the relationship, whereby cardiometabolic disease can also exacerbate oral inflammatory conditions. Methodological challenges—such as exposure misclassification, residual confounding, and reverse causation—are discussed alongside emerging solutions, including data linkage, multi-omics integration, and advanced analytic approaches. Harnessing large-scale data sources offers a powerful opportunity to reposition oral health within cardiometabolic disease research and prevention. Strategic integration of dental and medical data has the potential to inform precision public health approaches and support more holistic models of chronic disease prevention.

## Introduction

Oral diseases and cardiometabolic disorders represent two of the most prevalent and burdensome groups of non-communicable diseases worldwide (1). According to the Global Burden of Disease (GBD) Study, oral disorders, predominantly untreated dental caries, severe periodontitis, and tooth loss, affect more than 3.5 billion people globally, making them among the most prevalent health conditions across all age groups. Although oral diseases contribute minimally to mortality, they are consistently ranked by GBD as a leading cause of years lived with disability (YLDs), reflecting their substantial impact on functional limitation, pain, and quality of life (2, 3). In parallel, cardiometabolic diseases, particularly cardiovascular disease and type 2 diabetes, constitute the largest contributors to global mortality and disability-adjusted life years (DALYs) (4). GBD estimates indicate that cardiovascular disease alone accounts for approximately one-third of all global deaths annually and remains the leading cause of years of life lost (YLLs) worldwide, while diabetes contributes substantially to both YLLs and YLDs through its chronic complications and premature mortality. Collectively, cardiometabolic disorders account for a dominant share of global DALYs, underscoring their central role in the global non-communicable disease burden (5). Importantly, GBD analyses consistently demonstrate that both oral diseases and cardiometabolic disorders are strongly socially patterned, with disproportionately higher prevalence, disability, and premature mortality observed in socioeconomically disadvantaged populations (6).

Over the past two decades, epidemiologic and mechanistic evidence has increasingly linked poor oral health, particularly periodontitis, tooth loss, and chronic oral inflammation, to cardiometabolic outcomes such as type 2 diabetes, atherosclerotic cardiovascular disease, and adverse cardiometabolic biomarker profiles (7). However, despite the growing body of association studies, major uncertainties remain regarding directionality, causal inference, and the extent to which observed links reflect shared determinants (e.g., smoking, diet, socioeconomic position, health behaviors, access to care) vs. biologically meaningful pathways.

Recent advances in biobank infrastructure, record linkage, and publicly accessible population-based databases now enable a new generation of oral–cardiometabolic research. Large cohorts with long follow-up, high-dimensional phenotyping, repeated biomarker measurements, imaging, genetics, and linkage to hospitalization and mortality registries allow investigators to test hypotheses with improved statistical power and a broader range of analytic strategies (8, 9). These resources are particularly valuable for clarifying potential bidirectionality (oral-to-cardiometabolic and cardiometabolic-to-oral pathways), probing effect modification by age/sex/comorbidity, and evaluating whether oral health proxies predict incident cardiometabolic events independently of conventional risk factors. At the same time, secondary analyses using biobanks introduce distinct challenges, most notably exposure misclassification when oral health is measured by brief self-report items rather than clinical periodontal examinations. For example, in UK biobank, commonly used oral variables come largely from the touchscreen “mouth/teeth problems” questionnaire field rather than standardized periodontal charting, which can limit phenotype precision and complicate causal interpretation (10–12).

Accordingly, the purpose of this review is to (i) map the current landscape of biobanks and large-scale epidemiologic resources relevant to oral–cardiometabolic research; (ii) describe which oral exposures and cardiometabolic outcomes are captured, and how they are operationalized; (iii) highlight key methodological strengths and limitations of these resources for oral–systemic inference; and (iv) outline analytic opportunities, such as linkage across dental and medical records, biomarker integration, and genetic approaches that can move the field from association toward more credible mechanistic and translational insight.

### Methodological approach of the narrative review

This narrative review was undertaken to critically examine how biobanks and large-scale epidemiologic datasets have been used to study the association between oral health—particularly periodontitis and tooth loss—and cardiometabolic outcomes. The emphasis was placed on methodological considerations, data structure, and analytic implications rather than on exhaustive enumeration of all published studies.

Large-scale cohorts and biobanks were included based on their relevance to oral–cardiometabolic research, specifically: (i) population-based design with substantial sample size; (ii) availability of oral health information, whether derived from clinical examination, self-reported measures, or electronic health record and claims data; (iii) robust ascertainment of cardiometabolic outcomes through clinical measurements, registries, or longitudinal follow-up; and (iv) capacity for linkage, repeated measurements, or integration with biomarker, genetic, or multi-omics data. On this basis, the review focuses on major population biobanks, nationally representative examination surveys, and EHR-linked cohorts that collectively illustrate the diversity of oral exposure definitions and cardiometabolic outcome ascertainment strategies.

The supporting literature was identified through targeted searches of PubMed, Scopus, and Web of Science using combinations of keywords including oral health, periodontitis, tooth loss, biobanks, large-scale epidemiologic studies, cardiometabolic disease, type 2 diabetes, and cardiovascular disease. Priority was given to large cohort studies, methodologically informative analyses, and reviews that explicitly addressed exposure definition, bias, data linkage, or analytic challenges. Evidence was synthesized thematically, with particular attention to how oral health variables are operationalized across data platforms and how these choices influence interpretation, generalizability, and inference.

### Biobank and large-scale data landscape for oral–cardiometabolic research

Large-scale resources relevant to oral–cardiometabolic research can be conceptualized as three partially overlapping ecosystems: (1) population biobanks and cohort studies with extensive follow-up and linkage to health outcomes; (2) nationally representative health examination surveys that include standardized oral assessments; and (3) EHR-linked or claims-based databases that capture real-world healthcare utilization and diagnoses, increasingly augmented by genomics and biobank sampling. Each ecosystem offers different trade-offs between sample size, representativeness, exposure validity for oral health, and the depth and reliability of cardiometabolic outcome ascertainment (13).

Population biobanks (e.g., UK Biobank) provide exceptional scale and longitudinal outcome capture, typically through linkage to hospital episode statistics, death registries, and disease registries, alongside baseline risk factors, blood biomarkers, and increasingly multi-omics (14). Their main limitation for oral–systemic research is that oral health is often captured via brief self-report items (e.g., mouth/teeth problems, dentures, tooth loss proxies, oral symptoms) rather than clinical periodontal measures, which can introduce non-differential misclassification and bias associations toward the null or generate heterogeneous phenotypes across studies depending on how oral variables are defined. UK Biobank's “mouth/teeth dental problems” field is widely used and explicitly documented as self-reported, with multiple categories that investigators often combine to approximate periodontal disease phenotypes (e.g., bleeding gums, painful gums, loose teeth), but these constructs should be interpreted as symptom-based proxies rather than definitive periodontitis diagnoses. Nonetheless, the scale of such datasets supports sensitivity analyses, triangulation with biomarkers (e.g., inflammatory markers), and stratified models that would be impractical in smaller clinically phenotyped cohorts (15–17).

In contrast, nationally representative examination surveys such as NHANES are particularly valuable because they include standardized oral health assessments performed by calibrated examiners, alongside cardiometabolic phenotyping that may include anthropometrics, laboratory biomarkers, and clinically defined disease status (18). The key strength of this ecosystem is higher measurement validity for oral exposures compared with symptom-only surveys, enabling more defensible inference about periodontal status and tooth-level outcomes. A practical limitation is smaller sample size than population biobanks and more limited longitudinal follow-up for incident outcomes (depending on the wave and linkage availability), which can constrain analyses of rare endpoints or subgroup effects. However, for many mechanistic and risk-factor questions especially those involving inflammatory and metabolic biomarkers, these surveys provide a high-quality bridge between oral clinical measures and cardiometabolic profiling (19, 20).

A third ecosystem is rapidly expanding: EHR-linked biobanks and real-world databases in which cardiometabolic outcomes (diagnoses, medications, procedures, lab results) are captured in routine care, and oral health information may be accessible through linkage to dental records, dental claims, or integrated medical–dental systems (13, 21). These resources are particularly aligned with the research topic's emphasis on integrating historically siloed data streams and generating translational public health insights (21). Programs such as the NIH All of Us Research Program exemplify this direction by assembling large-scale participant data with EHR components and a research infrastructure intended for broad secondary analyses, although the availability and granularity of dental information can vary by site and data provenance (22). The major scientific advantage of EHR-based ecosystems is clinical realism and the potential to study care pathways, multimorbidity, medication effects, and health inequalities. The major scientific risk is heterogeneity in coding practices, incomplete capture of periodontal diagnoses in dentistry, and limited interoperability between dental and medical software, problems that have been recognized as persistent barriers to integrating oral health into broader population health analytics (23).

Across these ecosystems, the cardiometabolic outcome space is typically stronger than the oral exposure space. Cardiometabolic endpoints—incident type 2 diabetes, myocardial infarction, stroke, heart failure, hypertension, dyslipidemia, and cardiometabolic biomarker trajectories, are usually well captured via registries, hospitalization records, medication records, and laboratory data (24). Oral exposures are more variable: in some datasets they are symptom-based proxies (useful for large-scale hypothesis screening), while in others they include clinical periodontal parameters and tooth-level assessments (better for phenotypic validity but often smaller and less longitudinal). The central opportunity, therefore, lies in deliberate triangulation: combining the scale and outcome reliability of biobanks, the exposure validity of examination surveys, and the real-world relevance of EHR/claims data to converge on findings that are consistent across measurement frameworks (25). This landscape also creates an ideal platform for integrating genetics and other omics to strengthen causal arguments and clarify whether observed oral–cardiometabolic relationships reflect direct biological pathways, shared upstream risk, or bidirectional reinforcement over the life course.

### Oral health exposures in large-scale epidemiologic and biobank data

A central challenge in oral–cardiometabolic research using large-scale epidemiologic resources is the operationalization of oral health exposures (26). Unlike cardiometabolic outcomes which are typically defined using validated clinical diagnoses, biomarkers, hospital records, and medication data, oral health variables in population biobanks are often derived from self-reported questionnaires, administrative codes, or simplified clinical proxies. This asymmetry in measurement depth has important implications for exposure misclassification, effect estimation, and the interpretation of observed associations (27).

In many large biobanks, oral health is captured through brief self-report items embedded within baseline questionnaires. These commonly include questions on tooth loss, use of dentures, bleeding gums, painful or loose teeth, mouth ulcers, and general “mouth/teeth problems.” While such variables do not correspond directly to standardized periodontal diagnoses based on probing depth or clinical attachment loss, they nonetheless capture symptom-based manifestations of oral disease that may reflect cumulative inflammatory burden or advanced disease states. From an epidemiologic perspective, these measures are best conceptualized as *oral health proxies* rather than definitive clinical phenotypes (28, 29). Their principal advantage lies in feasibility and scale, enabling hypothesis generation and large-sample risk modeling that would otherwise be impossible. Their principal limitation lies in reduced specificity and potential non-differential misclassification, which may attenuate true associations or generate heterogeneity across studies depending on how variables are combined and interpreted (30).

To address these limitations, investigators increasingly apply harmonization strategies that group multiple oral symptom items into composite exposure definitions intended to approximate periodontal disease or poor oral health status. For example, combinations of bleeding gums, loose teeth, and painful gums have been used to construct symptom-based periodontal indices, while edentulism and denture use are often treated as markers of cumulative lifetime oral disease and treatment history (31, 32). Although imperfect, these constructs may capture clinically meaningful gradients of oral inflammatory exposure when interpreted cautiously and validated against biomarker or outcome patterns. Importantly, the validity of such proxies is context-dependent and may vary by age, socioeconomic position, access to dental care, and cultural norms regarding symptom reporting (31, 32).

In contrast to biobanks relying primarily on self-report, nationally representative examination surveys such as NHANES incorporate standardized oral examinations conducted by calibrated examiners, including periodontal probing, attachment loss assessment, and tooth-level evaluations. These surveys offer substantially greater measurement validity for oral exposures and allow more precise phenotyping of periodontal disease severity and extent. However, the trade-off is reduced sample size relative to population biobanks and, in some cases, limited longitudinal follow-up for incident cardiometabolic outcomes. As a result, such datasets are particularly valuable for mechanistic and biomarker-focused analyses, while larger biobanks are better suited for long-term risk prediction and rare outcome analyses (33, 34).

Administrative and EHR-linked datasets represent a third mode of oral exposure capture, typically relying on diagnostic codes, procedure codes, or dental claims. While these sources offer real-world relevance and the potential for longitudinal tracking, periodontal disease is often under-coded in routine dental practice, and coding practices may vary widely across providers and healthcare systems. Consequently, absence of a periodontal code does not necessarily imply absence of disease, introducing systematic misclassification that may bias associations in unpredictable directions (35, 36).

Taken together, these considerations underscore the importance of explicitly acknowledging how oral health is defined in large-scale studies and avoiding overinterpretation of proxy measures as clinical diagnoses. Rather than viewing imperfect oral exposure measurement as a fatal limitation, contemporary oral–cardiometabolic research increasingly adopts a triangulation approach: leveraging multiple datasets with complementary strengths, testing consistency of associations across different exposure definitions, and integrating oral proxies with inflammatory biomarkers, metabolic profiles, and genetic data. This strategy aligns with the broader movement in epidemiology toward convergence of evidence, recognizing that no single dataset is sufficient to resolve complex oral–systemic relationships in isolation.

To improve interpretability and analytic consistency, oral health variables captured across large-scale datasets should be viewed through an operational rather than diagnostic lens. As summarized in Table 1, different data ecosystems capture distinct aspects of oral health, ranging from clinically defined periodontal status in examination surveys to symptom-based proxies and treatment indicators in population biobanks and EHR-linked cohorts. Self-reported oral symptoms are best interpreted as markers of inflammatory periodontal risk or cumulative oral disease burden rather than as definitive diagnoses. Administrative and claims-based indicators primarily reflect documented or treated disease and healthcare utilization. Recognizing these distinctions allows researchers to align exposure definitions with appropriate research questions, apply sensitivity analyses across data sources, and avoid overinterpretation of proxy measures as clinical periodontal disease.

**Table 1:** Operational framework for oral health exposure definitions across large-scale epidemiologic data ecosystems.

Data ecosystem	Oral health variable type	Examples of variables captured	What this reasonably represents	Recommended analytic use-cases	Key limitations
Examination surveys (e.g., NHANES)	Standardized clinical periodontal measures	Probing depth, clinical attachment loss, bleeding on probing, tooth count	Clinically defined periodontal status and disease severity	Etiologic inference, biomarker correlation, validation of proxy measures	Smaller sample size; limited longitudinal follow-up for incident outcomes
Population biobanks (e.g., UK Biobank)	Self-reported oral symptoms and dentition proxies	Bleeding gums, loose teeth, painful gums, denture use, tooth loss	Symptom-based inflammatory periodontal risk or cumulative oral disease burden	Hypothesis generation, large-scale risk modeling, stratified and sensitivity analyses	Non-specific; exposure misclassification; cannot distinguish gingivitis vs. periodontitis
EHR- or claims-linked cohorts	Diagnostic and procedure codes	Periodontal diagnosis codes, scaling/root planing, extractions	Treated or documented periodontal disease and care utilization	Care-pathway analysis, longitudinal disease trajectories, health services research	Under-coding of periodontal disease; reflects access to care as well as disease
Hybrid/linked datasets	Combined self-report, clinical, and administrative data	Oral symptoms plus biomarkers, medications, dental procedures	Multidimensional oral health phenotype	Triangulation, mediation analysis, life-course modeling	Data heterogeneity; requires careful harmonization

### Data-source harmonisation across oral health measures

Across large-scale epidemiologic datasets, oral health is captured through heterogeneous modalities, including standardized clinical periodontal examinations (e.g., NHANES), symptom-based self-report items (e.g., bleeding gums, loose teeth, denture use in population biobanks), and administrative or claims-derived indicators of treated disease. For analytic consistency, these measures should be interpreted operationally rather than diagnostically: clinical examinations approximate periodontal status and severity; self-reported symptoms primarily reflect inflammatory periodontal risk or cumulative oral disease burden; and administrative records capture documented disease and healthcare utilization. Harmonization across data sources therefore requires alignment of exposure definitions with research objectives, application of sensitivity analyses using alternative proxies where available, and triangulation across complementary datasets to assess robustness of associations.

### Cardiometabolic outcomes captured in biobanks and population databases

In contrast to the relative heterogeneity and limitations of oral health measurement, cardiometabolic outcomes are generally well characterized in large-scale epidemiologic and biobank datasets (37, 38). Major cardiometabolic conditions including type 2 diabetes, hypertension, dyslipidemia, obesity, coronary heart disease, stroke, and heart failure are typically captured through a combination of self-report, clinical measurements, laboratory biomarkers, medication records, hospital admissions, and linkage to disease and mortality registries (37–39). This robust outcome ascertainment represents a key strength of biobank-based oral–cardiometabolic research and provides a stable foundation upon which oral exposure hypotheses can be tested.

Type 2 diabetes is among the most comprehensively documented cardiometabolic outcomes across large datasets, often defined using fasting glucose, HbA1c, physician diagnosis, medication use, or combinations thereof. Many biobanks also enable differentiation between prevalent and incident diabetes, facilitating longitudinal analyses of oral health as a potential risk factor for disease onset (40, 41). Cardiovascular outcomes such as myocardial infarction and stroke are commonly ascertained through validated hospital episode statistics and cause-of-death registries, which substantially reduces outcome misclassification compared with self-reported disease histories (42).

Beyond categorical disease endpoints, large-scale datasets increasingly include continuous cardiometabolic biomarkers that are particularly valuable for mechanistic inference. These include inflammatory markers (e.g., C-reactive protein), lipid profiles, glycemic indices, anthropometric measures, and blood pressure readings. Such variables allow investigators to explore whether oral health proxies are associated not only with overt disease but also with subclinical cardiometabolic dysregulation along a continuum of risk. This is especially relevant for oral health research, where chronic low-grade inflammation is hypothesized to exert systemic effects long before clinical cardiovascular events occur (43–45).

The relative strength of cardiometabolic outcome measurement also enables more sophisticated analytic approaches, including mediation analyses, effect modification assessments, and trajectory modeling. For example, investigators can examine whether the association between oral health proxies and cardiovascular events is mediated by inflammatory biomarkers, glycemic control, or lipid abnormalities, or whether associations differ by sex, age, or baseline metabolic status (26, 46, 47) (Box 1). While such analyses cannot establish causality in observational settings, they can provide valuable insight into plausible biological pathways and inform hypotheses for future interventional studies. Importantly, the asymmetry between strong cardiometabolic outcome data and weaker oral exposure data necessitates careful interpretation. Observed associations should not be construed as definitive evidence of oral disease causally driving cardiometabolic outcomes, particularly when oral exposures are measured crudely. Rather, the strength of cardiometabolic phenotyping should be leveraged to test consistency, dose–response patterns, and temporal relationships, thereby strengthening or weakening confidence in proposed oral–systemic links.

Box 1 **Strengths and limitations of large-scale oral–cardiometabolic research.**

**Table:** 

Strengths
• Large sample sizes enable detection of modest associations and subgroup analyses.
• Longitudinal linkage supports incident cardiometabolic outcome assessment.
• Cardiometabolic endpoints are typically well validated through registries and laboratory measures.
• Integration with genomics and biomarkers enables triangulation and mechanistic exploration.
Limitations
• Non-differential misclassification of oral health proxies, particularly symptom-based measures in population biobanks, is expected to attenuate effect estimates toward the null, with validation studies suggesting reductions in hazard ratios of approximately 15%–25%.
• Residual confounding by socioeconomic status, health behaviors, and access to dental care cannot be fully eliminated in observational datasets.
• Reverse causation and bidirectional relationships complicate temporal interpretation, particularly in cross-sectional or short follow-up analyses.
• Healthy volunteer bias limits external validity, as participants in large biobanks tend to be healthier and more healthcare-engaged than the general population.
• Under-coding of periodontal disease in electronic health records conflates disease burden with healthcare utilization, contributing to differential exposure misclassification

### Major biobanks and population databases enabling oral–cardiometabolic research

The rapid expansion of large-scale biobanks and population-based cohorts has reshaped oral–cardiometabolic research by enabling analyses at unprecedented scale while introducing substantial heterogeneity in exposure measurement, population composition, and outcome ascertainment. These resources differ markedly in study design, depth of phenotyping, and linkage capacity, underscoring the importance of aligning research questions with dataset-specific strengths. Rather than relying on any single cohort as definitive, contemporary oral–systemic research increasingly adopts a multi-database strategy, leveraging complementary data ecosystems to triangulate associations and enhance interpretability (8, 48) (Table 2).

**Table 2:** Comparison of oral health, biospecimen availability, and cardiometabolic data capture across major population-based cohorts.

Cohort	Region	Approx Sample Size	Oral Health Assessment	Periodontal Phenotyping	Biospecimens Available	Cardiometabolic Outcomes	Study Design/Follow-Up	Key Strengths
UK Biobank	United Kingdom	∼500,000	Self-reported oral indicators (tooth loss, oral ulcers); no routine clinical periodontal exams	Limited (proxy measures only)	Blood, serum, plasma, urine, DNA; imaging; no routine saliva	CVD, diabetes, hypertension, lipid traits, imaging phenotypes	Prospective cohort with linkage to EHR and death registries	Massive scale, deep phenotyping, genomics, long-term outcomes; ideal for causal inference (e.g., Mendelian randomization)
NHANES	United States	∼10,000 per cycle	Comprehensive clinical dental exams; periodontal probing in several cycles	High (full- or partial-mouth periodontal measures)	Blood, urine, DNA (selected cycles); limited saliva	Glucose, HbA1c, lipids, BP, obesity indices; mortality linkage	Repeated cross-sectional with longitudinal mortality follow-up	Gold-standard clinical oral data paired with rich metabolic biomarkers
All of Us Research Program	United States	>400,000 (growing)	Mainly self-reported; dental EHR integration evolving	Currently limited; improving via EHR	Blood, urine, saliva, DNA, EHR-linked labs	EHR-derived cardiometabolic diagnoses, labs, genomics	Prospective longitudinal	High ethnic diversity, real-world clinical data, multi-omics
EPIC Study	Europe (10 countries)	∼520,000	Center-dependent; mostly self-reported oral indicators	Moderate and heterogeneous	Blood, serum, plasma, DNA (subset saliva)	Incident CVD, diabetes; extensive dietary biomarkers	Prospective cohort	Exceptional nutrition data and long-term chronic disease follow-up
China Kadoorie Biobank	China	∼512,000	Self-reported oral health (e.g., tooth loss)	Low (no standardized probing)	Blood, plasma, DNA	Stroke, ischemic heart disease, diabetes	Prospective cohort with registry linkage	Large East Asian population, high cardiometabolic event rates, long follow-up

Among these resources, the UK Biobank has been particularly influential due to its large sample size, longitudinal follow-up, and robust ascertainment of cardiometabolic outcomes, including incident diabetes, cardiovascular events, and mortality. Integration of inflammatory and metabolic biomarkers, imaging data, and genome-wide genotyping further supports multidimensional analyses that extend beyond conventional epidemiologic modeling (49–52). In this context, UK Biobank is especially well suited for large-scale hypothesis generation, risk stratification, and sensitivity analyses, while detailed periodontal phenotyping is limited by reliance on symptom-based oral health proxies.

NHANES offers a complementary model through nationally representative sampling combined with standardized clinical periodontal examinations and comprehensive cardiometabolic phenotyping. Its principal contribution lies in the high validity of oral health measurements and availability of laboratory biomarkers, enabling mechanistic exploration and validation of exposure definitions applied in larger biobanks. Although NHANES is constrained by smaller sample size and more limited longitudinal follow-up, linkage to mortality and selected hospitalization data partially mitigates these limitations, positioning it as an important phenotypic reference for oral–cardiometabolic research (19, 53).

The All of Us Research Program represents an evolving infrastructure explicitly designed to integrate electronic health records, surveys, physical measurements, biospecimens, and genomic data, with a strong emphasis on diversity and inclusion. Cardiometabolic outcomes are captured longitudinally through EHR linkage, while dental data availability remains heterogeneous, reflecting persistent structural separation between medical and dental care systems. Nevertheless, All of Us exemplifies the future direction of oral–systemic research, in which cross-domain linkage enables investigation of real-world care pathways, multimorbidity, and health disparities at scale (54, 55).

Large European cohorts such as EPIC and major Asian resources including the China Kadoorie Biobank further extend the geographic and sociocultural scope of oral–cardiometabolic research. These datasets provide substantial sample sizes, long follow-up, and detailed lifestyle and cardiometabolic outcome information, supporting cross-population comparisons. Although oral health measures in these cohorts are often limited or indirect, their inclusion is critical for assessing whether observed associations are consistent across populations with differing risk profiles, healthcare infrastructures, and social determinants of health (56, 57).

Across these diverse resources, cardiometabolic outcomes are generally captured with greater precision and longitudinal depth than oral health exposures, reflecting historical underinvestment in oral health surveillance and continued fragmentation between dental and medical data systems. At the same time, this imbalance highlights a key opportunity: by combining the scale and outcome reliability of population biobanks, the exposure validity of examination surveys, and the clinical realism of EHR-linked datasets, investigators can triangulate evidence and mitigate limitations inherent to any single platform (58–62). The expanding availability of genetic data further broadens analytic possibilities, enabling Mendelian randomization, polygenic risk scoring, and gene–environment interaction analyses to explore shared susceptibility and potential directional relationships between oral and cardiometabolic conditions. While these approaches remain subject to methodological assumptions, they represent an important advance in a field historically dominated by cross-sectional associations (63, 64).

In the two-sample bidirectional Mendelian randomization analysis by Li et al., genetic instruments for periodontitis were derived from large European GWAS datasets (*n* = 198,441 and *n* = 195,762), with coronary atherosclerosis outcomes obtained from 61,194 cases; independent single-nucleotide polymorphisms were selected at genome-wide significance (*P* < 5×10⁻⁸) and analyzed primarily using inverse-variance weighted models (65).

In contrast, MR and integrative analyses incorporating multi-omics data reported that genetically predicted periodontitis was associated with increased risk of type 2 diabetes and insulin resistance (OR ≈ 1.47; 95% CI 1.12–1.93; *P* = 0.006), suggesting shared genetic susceptibility between oral inflammation and metabolic dysregulation (66).

Additional MR studies within related oral phenotypes highlight heterogeneous findings, underscoring the need for stronger periodontal genetic instruments and rigorous analytic triangulation (67, 68).

An updated scientific statement from the American Heart Association, *Periodontal Disease and Atherosclerotic Cardiovascular Disease*, synthesizes current evidence on the association between periodontal disease and atherosclerotic cardiovascular disease (ASCVD) and updates the AHA's 2012 statement on this subject. The document highlights that periodontal disease is highly prevalent in adults and is consistently associated with multiple ASCVD outcomes, including coronary heart disease, stroke, and peripheral artery disease, even after accounting for shared risk factors such as smoking, diabetes, and hypertension. It emphasizes potential biological mechanisms including chronic systemic inflammation and microbial translocation, and calls for further research to clarify whether periodontal treatment modifies ASCVD risk. While the statement supports a consistent association between periodontal disease and ASCVD, it also notes that definitive causal effects and impacts of periodontal therapy on hard cardiovascular outcomes remain to be established. This expert consensus underscores the importance of integrating oral health into broader cardiovascular risk assessment and prevention strategies (69).

Collectively, the current generation of biobanks and large-scale epidemiologic databases offers an unprecedented opportunity to refine population-level understanding of oral–cardiometabolic relationships. Realizing this potential requires explicit attention to how oral exposures are operationalized, strategic use of complementary datasets, and cautious interpretation that avoids overstating causality. When applied thoughtfully, these resources can move the field toward a more integrated and methodologically robust framework in which oral health is examined as a component of broader cardiometabolic risk.

To translate the database ecosystem perspective into a practical research guide, Table 3 summarizes key biobanks and large-scale cohorts commonly used in oral–cardiometabolic research, highlighting how oral health and cardiometabolic variables are captured, the longitudinal and linkage structure of each resource, and their primary strengths and limitations. This consolidated roadmap is intended to support alignment between research questions, exposure definitions, and analytic strategies, and to facilitate triangulation across complementary data ecosystems.

**Table 3:** Roadmap of major biobanks and large-scale cohorts for oral–cardiometabolic research.

Cohort/Biobank	Oral health variable type	Cardiometabolic outcomes	Longitudinal/linkage structure	Key strengths	Key limitations & recommended use-cases
UK Biobank	Self-reported oral symptoms, denture use, tooth loss proxies	Incident type 2 diabetes, CVD events, cardiometabolic biomarkers, mortality	Long-term follow-up with hospital and death registry linkage	Very large sample size; robust cardiometabolic outcome ascertainment; biomarker and genetic data	Oral health measures are symptom-based proxies; best suited for hypothesis generation, risk stratification, and sensitivity analyses
NHANES	Standardized clinical periodontal examination, tooth-level assessments	Diabetes status, glycemic and lipid biomarkers, blood pressure, mortality linkage	Repeated cross-sectional surveys with limited longitudinal linkage	High validity of oral phenotyping; rich biomarker data	Smaller sample size; limited incident outcome follow-up; ideal for validation and mechanistic analyses
All of Us Research Program	Heterogeneous (EHR-linked dental data, self-report where available)	Diabetes, CVD, medications, laboratory results	Ongoing EHR linkage; expanding longitudinal depth	Emphasis on diversity; integrated medical data	Dental data completeness varies by site; suitable for health disparities and real-world analyses
EPIC Study	Limited or indirect oral health indicators	Incident diabetes, CVD, mortality	Long-term prospective follow-up	Geographic diversity; strong dietary and lifestyle data	Oral health data limited; useful for cross-context consistency analyses
China Kadoorie Biobank	Limited self-reported oral health indicators	Cardiovascular disease, diabetes, mortality	Very large population-based cohort with long follow-up	Representation of non-Western populations; high external relevance	Sparse oral phenotyping; useful for transportability and population-level inference
EHR/claims-linked cohorts	Diagnostic codes, dental procedures, claims data	Cardiometabolic diagnoses, medications, healthcare utilization	Real-world longitudinal care pathways	Clinical realism; treatment trajectory analysis	Under-coding of periodontal disease; reflects access to care as well as disease

### Biological mechanisms linking oral health and cardiometabolic disease

While the biological mechanisms linking oral health and cardiometabolic disease have been extensively characterized in experimental and clinical research, their relevance within large-scale epidemiologic and biobank studies depends on the extent to which these pathways can be approximated using available population-level data. Accordingly, the mechanisms discussed below are framed not only in terms of biological plausibility, but also in relation to intermediate phenotypes, biomarkers, and clinical variables that are commonly captured in biobanks and large-scale datasets. This “database lens” allows mechanistic hypotheses to be evaluated indirectly through measurable inflammatory, metabolic, microbial, and vascular indicators, even in the absence of detailed periodontal phenotyping**.**

**Figure 1:**
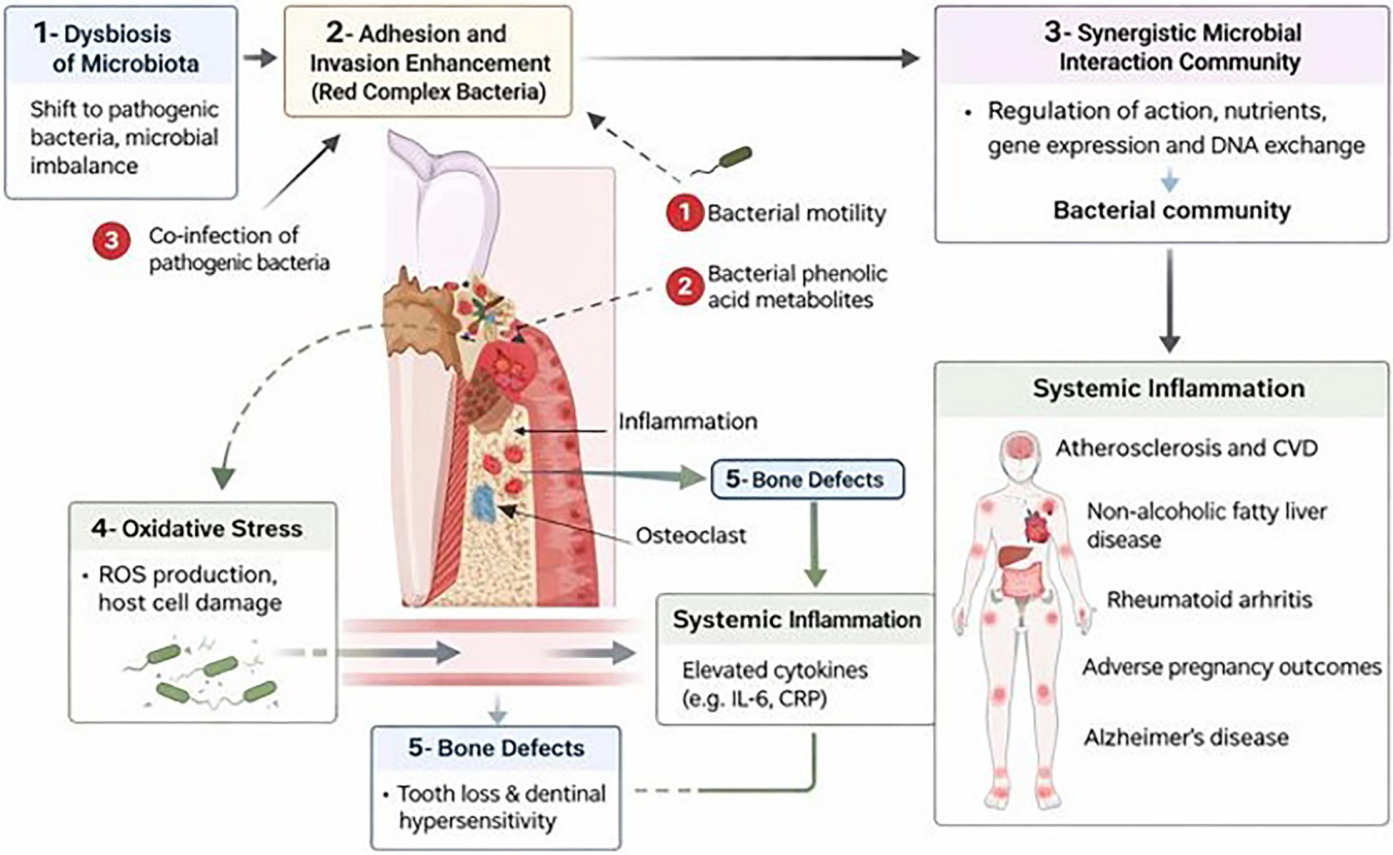
Conceptual diagram showing how periodontal dysbiosis triggers local inflammation and bone loss while systemic inflammatory signaling links oral infection to cardiometabolic disease (3).

The association between poor oral health and cardiometabolic disease is biologically plausible and supported by converging evidence from immunology, microbiology, vascular biology, and metabolic research. Periodontitis, the most extensively studied oral exposure in this context, represents a chronic inflammatory condition characterized by persistent microbial dysbiosis, epithelial barrier disruption, and sustained host immune activation (70). These features position periodontal disease as a potential contributor to systemic cardiometabolic dysregulation through multiple, partially overlapping pathways rather than a single linear mechanism (Figure 1).

While the biological pathways linking oral inflammation to cardiometabolic disease are well characterized experimentally, their evaluation in population-scale datasets depends on the availability of measurable intermediate phenotypes and proxy variables. To align mechanistic hypotheses with the realities of biobank and cohort data structures, it is essential to explicitly map each major pathway to observable markers captured through laboratory testing, questionnaires, electronic health records, medication data, imaging, and omics sub-studies. Table 4 provides a structured framework linking key oral–cardiometabolic mechanisms to candidate intermediate phenotypes and indicates their availability across major data platforms, together with common methodological limitations such as exposure misclassification, timing discordance, and under-coding.

**Table 4:** Mapping of mechanistic pathways to measurable proxies across large-scale data platforms.

Major pathway	Candidate intermediate phenotypes/proxies	Typical data source	Availability by platform	Common limitations
Chronic systemic inflammation	CRP, leukocyte count, IL-6 (subset), fibrinogen	Laboratory biomarkers	Exam surveys (NHANES): Yes; Population biobanks (UK Biobank/EPIC): Yes; EHR/claims: partial; Omics sub-cohorts: expanded	Single time-point measurement, temporal discordance with oral exposure, residual confounding
Metabolic dysregulation/insulin resistance	Fasting glucose, HbA1c, insulin, lipid profile, BMI, waist circumference	Labs + physical measures	Exam surveys: Yes; Biobanks: Yes; EHR/claims: Yes; Omics: metabolomics in subsets	Medication effects, variable fasting status, missingness
Vascular dysfunction/atherosclerosis	Blood pressure, incident MI/stroke, imaging-derived arterial measures (subset)	EHR/registries, imaging	Biobanks: Yes (events + imaging subsets); EHR/claims: Yes; Exam surveys: limited	Hard endpoints lag exposure; imaging only in sub-cohorts
Microbial translocation/dysbiosis (indirect)	CRP, WBC, endotoxemia-related markers; microbiome only in sub-studies	Biomarkers/omics	Routine platforms: indirect only; Omics sub-cohorts: oral/gut microbiome	Rare availability of direct oral microbiome; proxy-based inference
Shared genetic susceptibility	Polygenic risk scores, GWAS variants	Genomics	Population biobanks: Yes; Exam surveys: limited; EHR-linked biobanks: Yes	Weak periodontal instruments; pleiotropy
Medication-mediated pathways	Antidiabetics, statins, antihypertensives, antiplatelets	Pharmacy/EHR records	Biobanks with EHR: Yes; Claims databases: Yes; Exam surveys: limited	Indication bias; adherence unknown
Cumulative oral disease burden	Tooth loss, denture use, oral symptom composites	Self-report/dental records	Biobanks: Yes; Exam surveys: clinical tooth counts; EHR/claims: procedures	Non-specific proxies; under-coding of periodontitis
Socio-behavioral modifiers	Smoking, diet, physical activity, SES indices	Questionnaires/registries	Exam surveys + biobanks: Yes; EHR: partial	Measurement error; residual confounding

### Example analysis path 1 (inflammatory mediation)

Self-reported periodontal symptoms or tooth loss (oral exposure, population biobank) → circulating CRP and leukocyte count (laboratory biomarkers) → incident cardiovascular disease identified through hospital records. This pathway allows testing whether systemic inflammation partially mediates the association between oral health proxies and cardiovascular events, acknowledging timing discordance between baseline oral measures and later outcomes.

### Example analysis path 2 (metabolic pathway)

Oral disease proxy (tooth loss or denture use) → HbA1c/fasting glucose and lipid profile (biobank laboratory data) → incident type 2 diabetes or cardiometabolic multimorbidity captured via EHR linkage. This framework supports evaluation of whether poor oral health is associated with progressive metabolic dysregulation prior to overt diabetes diagnosis.

### Example analysis path 3 (genetic triangulation)

Genetically predicted periodontitis (GWAS instruments) → inflammatory/metabolic biomarkers → cardiometabolic outcomes, enabling triangulation between observational associations and Mendelian randomization estimates.

**Figure 2:**
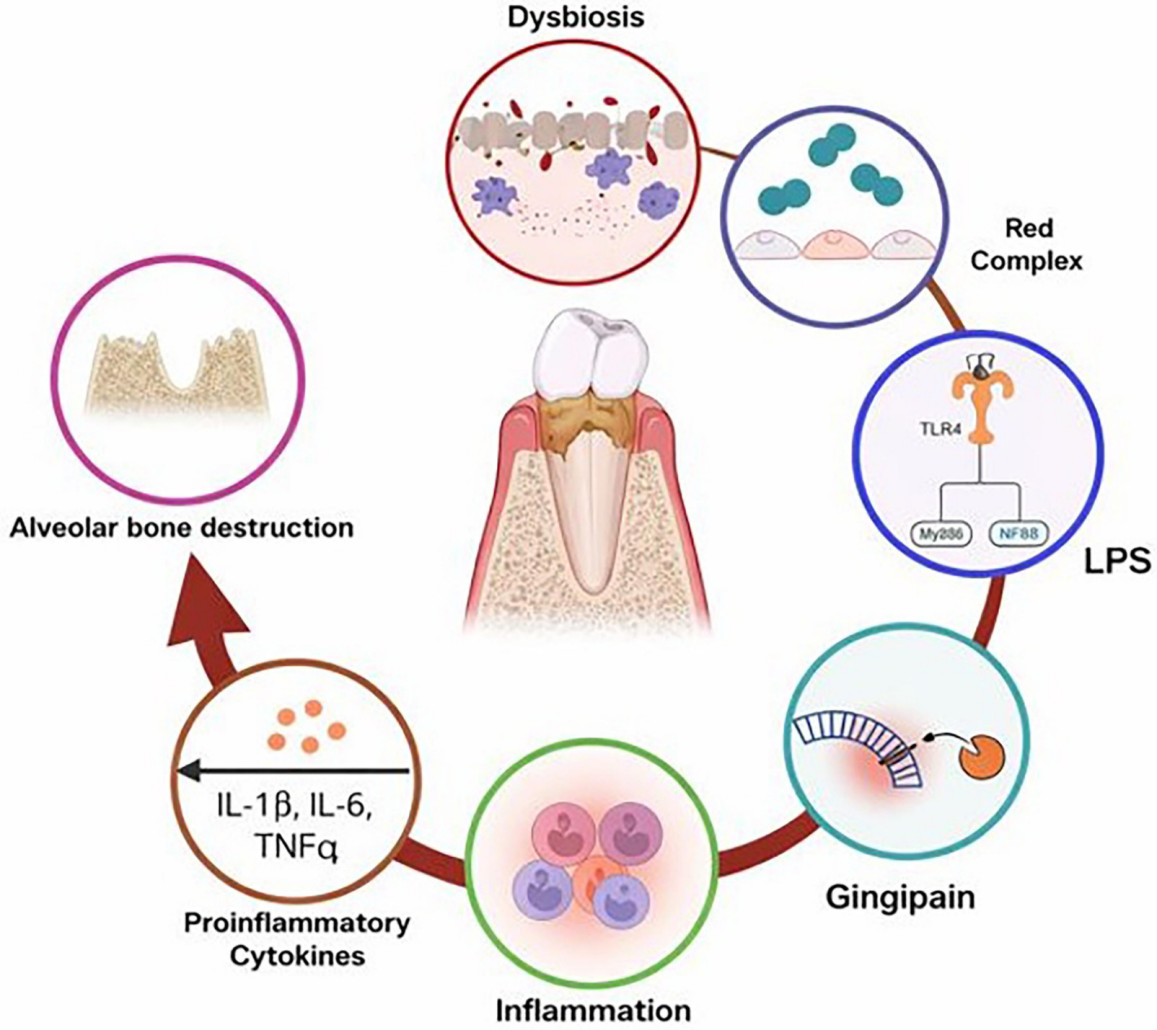
Schematic illustrating activation of host immune responses by periodontal pathogens, leading to cytokine release and inflammatory tissue destruction (72).

A central mechanistic link lies in chronic low-grade systemic inflammation, a shared hallmark of both periodontitis and cardiometabolic disorders. Periodontal inflammation is driven by a dysbiotic biofilm dominated by Gram-negative anaerobes that stimulate host immune responses through pathogen-associated molecular patterns, including lipopolysaccharides and other virulence factors. Local production of pro-inflammatory mediators such as interleukin-1β, interleukin-6, tumor necrosis factor-α, and prostaglandin E2 does not remain confined to the periodontal tissues (71) (Figure 2). Instead, these mediators can spill over into the systemic circulation, contributing to a sustained inflammatory milieu that promotes insulin resistance, endothelial dysfunction, and atherogenesis. Elevated circulating inflammatory markers observed in individuals with severe periodontal disease mirror those implicated in the pathophysiology of type 2 diabetes and cardiovascular disease, supporting inflammation as a biologically coherent bridge between oral and systemic pathology (73).

In large-scale datasets, this inflammatory pathway can be interrogated using circulating biomarkers such as C-reactive protein, interleukin-6, leukocyte counts, and other acute-phase reactants, which are widely available in population biobanks and enable evaluation of whether oral health proxies are associated with systemic inflammatory burden.

**Figure 3:**
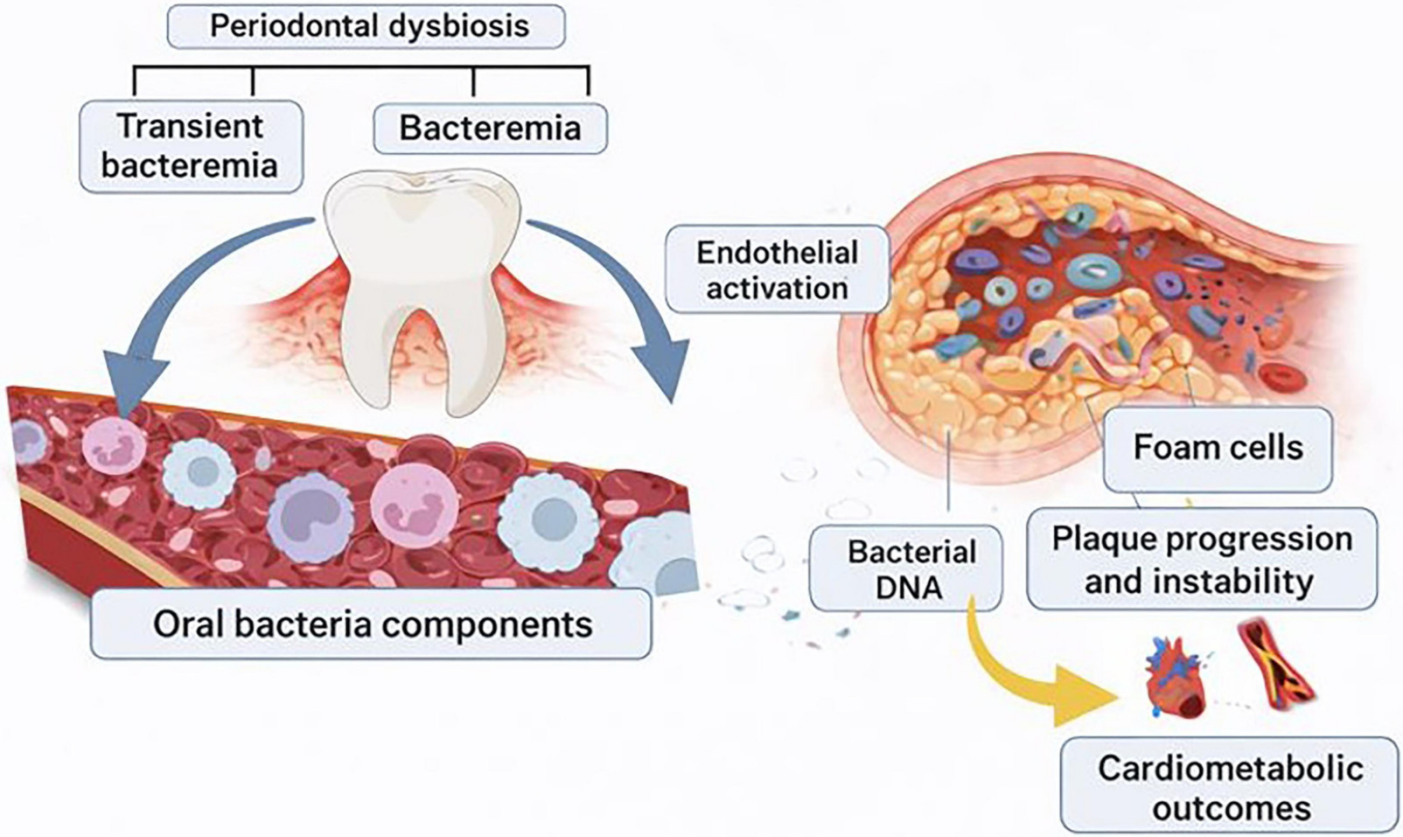
Diagram depicting transient bacteremia from periodontal tissues causing endothelial activation, plaque progression, and vascular inflammation. Created with Biorender.com.

Beyond soluble inflammatory mediators, periodontal dysbiosis and transient bacteremia represent an additional mechanistic pathway linking oral disease to cardiometabolic outcomes. Routine activities such as tooth brushing, flossing, or mastication can facilitate the entry of oral bacteria and their structural components into the systemic circulation, particularly in the presence of inflamed, ulcerated periodontal epithelium. Periodontal pathogens and their molecular signatures have been detected within atherosclerotic plaques and vascular tissues, supporting the concept that oral microbes may directly interact with the vascular endothelium. Such interactions can enhance endothelial activation, amplify local inflammatory signaling, promote foam cell formation, and contribute to plaque progression and instability (74, 75) (Figure 3). Although identification of bacterial DNA within vascular lesions does not in itself demonstrate causality, it reinforces the concept of the oral cavity as a chronic source of microbial products and inflammatory stimuli with potential relevance to systemic vascular biology. In large epidemiologic and biobank settings, direct microbial detection is rarely available; however, downstream vascular correlates of periodontal bacteremia can be explored indirectly through cardiometabolic endpoints, imaging-based markers of atherosclerosis where available, and inflammatory or endothelial biomarkers captured in biobank sub-studies.

**Figure 4:**
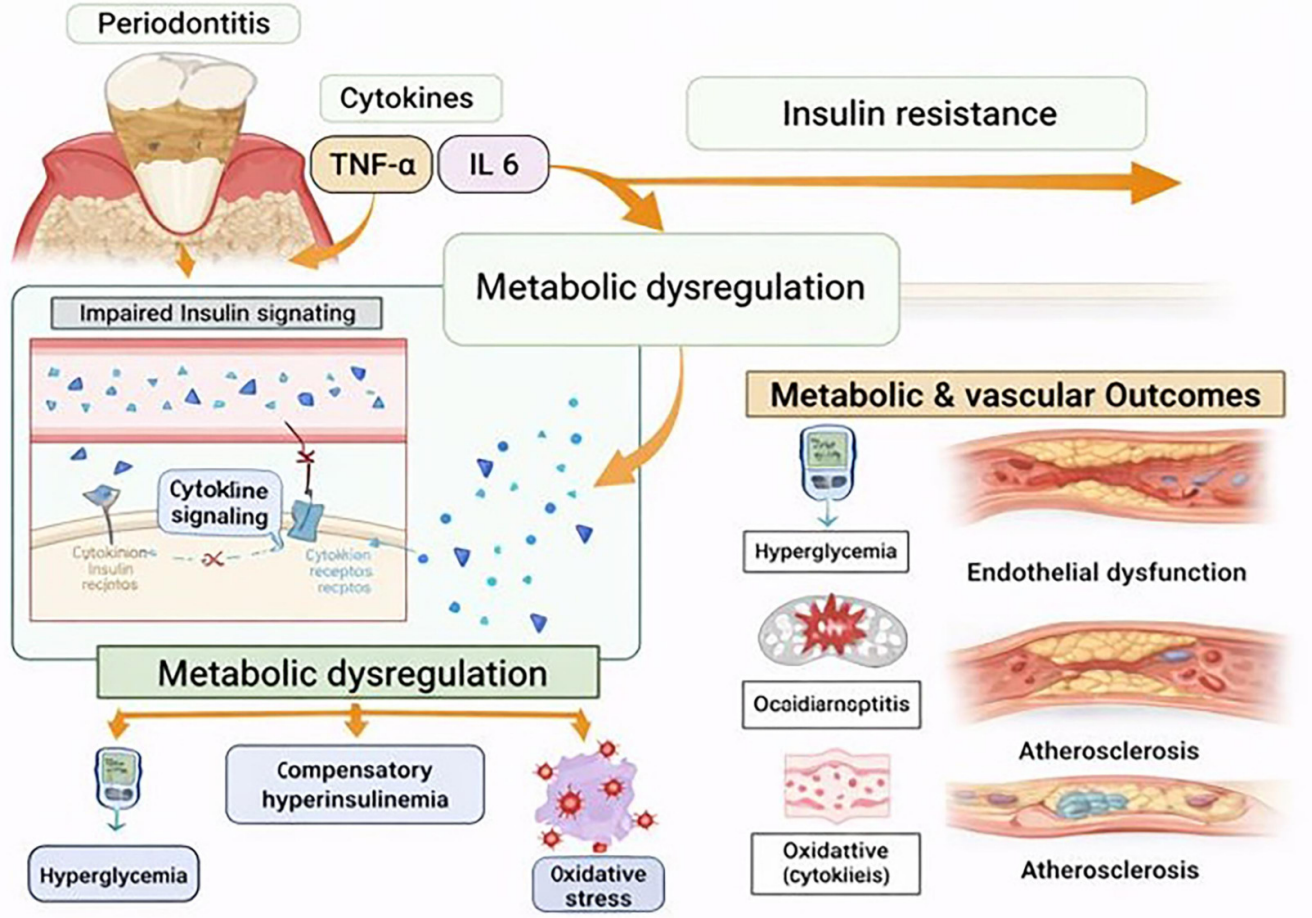
Pathway showing cytokine-mediated impairment of insulin signaling linking periodontitis with metabolic and vascular dysfunction. Created with Biorender.com.

Metabolic dysregulation, particularly insulin resistance, represents another critical convergence point. Periodontal inflammation can interfere with insulin signaling pathways through cytokine-mediated mechanisms, notably via tumor necrosis factor-α–induced serine phosphorylation of insulin receptor substrates (76). This impairment of insulin signaling contributes to hyperglycemia and compensatory hyperinsulinemia, which in turn exacerbate oxidative stress and vascular injury (76) (Figure 4). Importantly, large-scale epidemiologic studies consistently demonstrate that individuals with poor oral health exhibit worse glycemic control and higher risk of incident type 2 diabetes, even after adjustment for conventional risk factors. These observations align with mechanistic data suggesting that periodontal inflammation can worsen metabolic homeostasis, creating a feed-forward loop between oral and systemic disease (77, 78).

This pathway is particularly amenable to investigation in large-scale datasets, where fasting glucose, HbA1c, insulin levels, lipid profiles, and medication use are routinely measured, allowing assessment of whether oral health proxies are associated with metabolic dysfunction along a continuum rather than only with overt disease.

**Figure 5:**
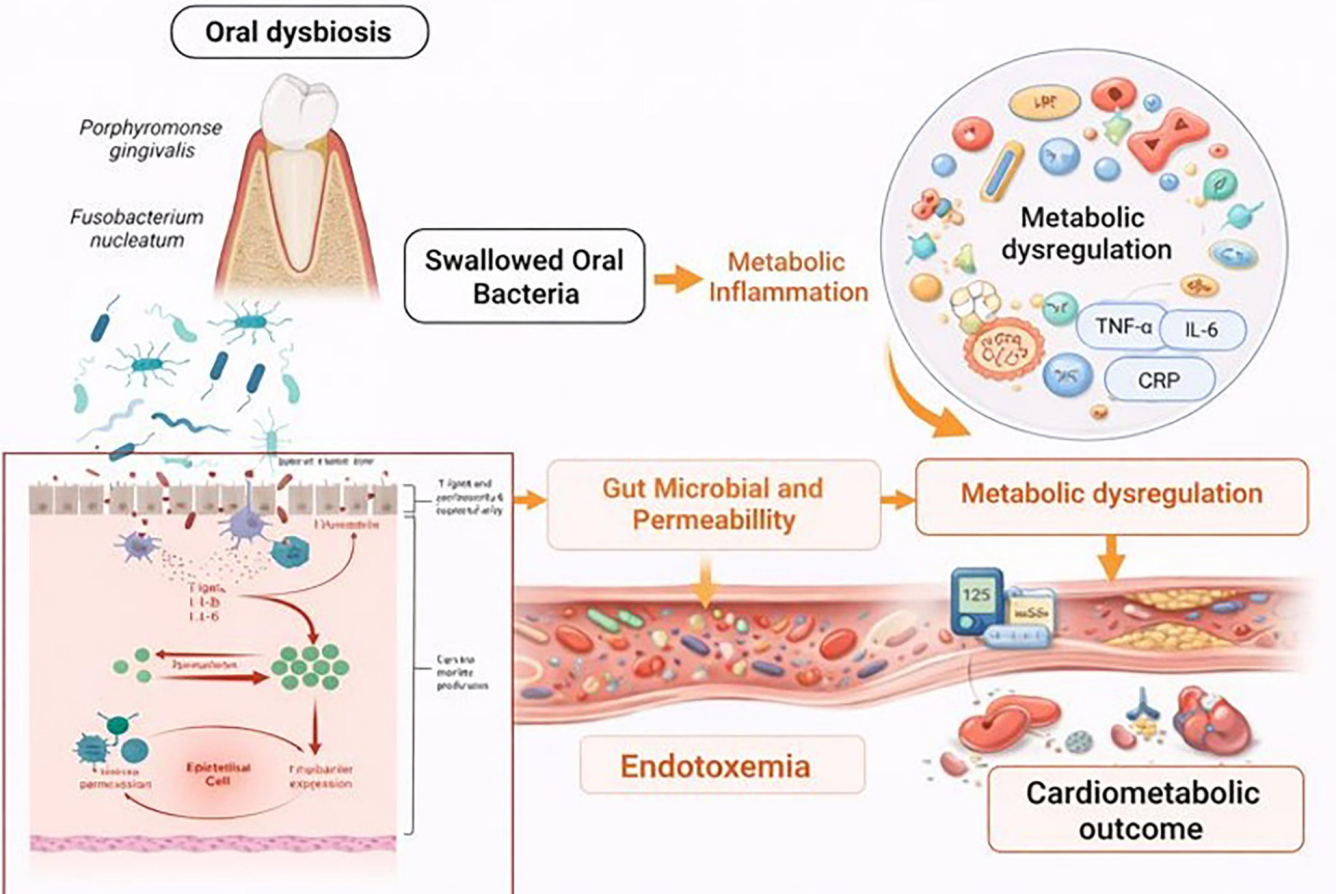
Illustration of oral–gut microbiome interactions contributing to metabolic inflammation and cardiometabolic risk. Created with Biorender.com.

The oral microbiome–host metabolic axis has emerged as an additional layer of complexity in recent years. Oral dysbiosis may influence systemic metabolism not only through direct inflammatory signaling but also via modulation of gut microbiota composition following swallowed oral bacteria (79) (Figure 5). Experimental and observational data suggest that oral pathogens can alter gut microbial ecology, intestinal permeability, and metabolic signaling, thereby contributing to systemic endotoxemia and metabolic inflammation. This concept broadens the oral–cardiometabolic connection beyond local periodontal tissues, situating the oral cavity within a larger network of host–microbiome interactions relevant to cardiometabolic risk (80–82).

While comprehensive microbiome profiling is available only in selected sub-cohorts, large biobanks increasingly include metabolomic signatures, inflammatory markers, and gastrointestinal phenotypes that can serve as indirect indicators of microbiome-mediated metabolic effects.

Endothelial dysfunction provides a unifying vascular mechanism linking oral inflammation to cardiovascular disease. Pro-inflammatory mediators and oxidative stress associated with periodontitis impair endothelial nitric oxide bioavailability, disrupt vascular homeostasis, and promote leukocyte adhesion and smooth muscle proliferation. These processes are central to the initiation and progression of atherosclerosis (83). Observational studies within large cohorts frequently report associations between poor oral health proxies and markers of vascular dysfunction, supporting the relevance of this pathway at the population level (84, 85).

At the population level, endothelial dysfunction related to oral inflammation may be approximated using blood pressure trajectories, vascular biomarkers, and incident cardiovascular events captured through longitudinal follow-up and registry linkage in biobanks.

Importantly, emerging evidence also points to shared genetic and epigenetic susceptibility as a contributor to oral–cardiometabolic comorbidity. Genetic variants influencing immune regulation, inflammatory responses, and metabolic pathways may predispose individuals to both periodontal disease and cardiometabolic disorders. Epigenetic modifications induced by chronic inflammation, smoking, or metabolic stress may further reinforce this overlap by altering gene expression patterns in immune and vascular cells. The availability of genome-wide data within large biobanks provides an unprecedented opportunity to explore these shared susceptibilities and to distinguish causal effects from correlated risk (86–88).

Collectively, these interrelated pathways support the interpretation of poor oral health as both a correlate and a potential modifier of cardiometabolic risk, rather than as an isolated causal driver. The oral–cardiometabolic interface is more appropriately conceptualized as a dynamic network of interacting inflammatory, microbial, metabolic, vascular, and genetic processes operating across the life course. Within this framework, large-scale epidemiologic and biobank studies—despite limited resolution of periodontal phenotypes—provide a unique opportunity to examine whether mechanistically plausible associations manifest as consistent population-level patterns across diverse settings. When considered alongside experimental and clinical evidence, such population-based observations help contextualize oral health within the broader architecture of cardiometabolic disease development and progression. Moreover, these datasets enable evaluation of intermediate phenotypes that link molecular pathways to clinical outcomes, facilitating convergence between biological plausibility and epidemiologic signal.

### Bidirectionality: cardiometabolic disease as a determinant of oral health

Emerging evidence supports a bidirectional association between oral health and cardiometabolic conditions, whereby periodontal inflammation and metabolic dysregulation appear to co-evolve over time rather than follow a strictly unidirectional pathway. While experimental and clinical studies suggest biological plausibility for reciprocal interactions, large-scale epidemiologic data primarily capture temporal associations rather than causality. Accordingly, observed relationships in population datasets should be interpreted as reflecting dynamic interdependence shaped by shared inflammatory, behavioral, and socioeconomic determinants (89–91).

Type 2 diabetes represents the most extensively studied cardiometabolic condition influencing oral health. Chronic hyperglycemia alters host immune function through multiple mechanisms, including impaired neutrophil chemotaxis and phagocytosis, dysregulated cytokine production, and increased formation of advanced glycation end products. These changes compromise host defense against periodontal pathogens and amplify inflammatory tissue destruction. In addition, hyperglycemia adversely affects collagen metabolism and angiogenesis, resulting in impaired wound healing and reduced regenerative capacity of periodontal tissues (92, 93).

Large population-based studies consistently demonstrate higher prevalence and severity of periodontitis among individuals with diabetes, as well as worse periodontal outcomes in those with poor glycemic control. Within biobank settings, where diabetes status and glycemic biomarkers are robustly captured, these relationships provide a clear example of cardiometabolic disease shaping oral health trajectories (94, 95).

Obesity and metabolic syndrome further contribute to oral inflammatory burden through systemic low-grade inflammation and adipokine dysregulation. Adipose tissue–derived mediators such as leptin, resistin, and pro-inflammatory cytokines influence immune responses within the periodontium, potentially lowering the threshold for inflammatory activation in response to microbial challenge. Insulin resistance, a core feature of metabolic syndrome, may also impair periodontal tissue homeostasis by disrupting energy metabolism and oxidative balance in resident cells (96). Epidemiologic analyses frequently report associations between higher body mass index, central adiposity, or metabolic syndrome components and markers of poor oral health, even after accounting for shared behavioral risk factors (97, 98). These findings underscore the importance of considering cardiometabolic status as an upstream determinant of oral disease rather than merely a downstream consequence.

Cardiovascular disease and its pharmacologic management can also indirectly influence oral health. Medications commonly used in cardiometabolic care, including antihypertensives, antiplatelet agents, and lipid-lowering therapies, may affect salivary flow, gingival tissues, or bleeding risk, thereby modifying oral disease presentation and detection. Reduced salivary secretion, for example, can increase susceptibility to oral microbial imbalance and mucosal inflammation. In large-scale datasets, where medication use is often well documented, failure to account for these effects may confound observed associations between oral health proxies and cardiometabolic outcomes (99–101).

Importantly, cardiometabolic disease often coexists with functional, behavioral, and socioeconomic factors that further shape oral health outcomes (26). Individuals with advanced cardiometabolic disease may experience reduced mobility, competing health priorities, or limited access to dental care, all of which can contribute to delayed diagnosis and progression of oral disease. In biobank and EHR-linked studies, these contextual factors are variably captured and may partially mediate or confound observed relationships (102, 103). Their influence highlights the need to interpret oral–cardiometabolic associations within a broader life-course and health-systems framework rather than attributing effects solely to biological mechanisms.

From a methodological perspective, bidirectionality presents a major challenge for observational research using large-scale datasets. Cross-sectional analyses are particularly vulnerable to reverse causation, while even longitudinal studies may struggle to disentangle reciprocal effects when both oral and cardiometabolic conditions evolve gradually over time (104, 105). Advanced analytic approaches, such as time-updated exposure models, lagged analyses, and genetic instrumental variable methods, can partially address these issues, but careful conceptual framing remains essential. Rather than seeking to establish a single dominant direction of effect, many contemporary investigators increasingly conceptualize oral and cardiometabolic diseases as mutually reinforcing components of a shared inflammatory and metabolic network.

Recognizing cardiometabolic disease as a determinant of oral health has important implications for both research and clinical practice. It reinforces the need for integrated care models in which oral health is not isolated from chronic disease management, and it cautions against simplistic causal narratives that position periodontal disease solely as an independent risk factor for cardiometabolic outcomes. In the context of biobank-based research, explicit acknowledgment of bidirectionality strengthens the credibility of findings and aligns oral–systemic investigations with modern epidemiologic thinking.

### Methodological challenges and bias in large-scale oral–cardiometabolic research

Despite the unprecedented opportunities afforded by biobanks and large-scale epidemiologic datasets, research at the intersection of oral and cardiometabolic health is subject to a range of methodological challenges that complicate causal interpretation. Many of these limitations stem from the historical marginalization of oral health within population health surveillance systems and the consequent imbalance between detailed cardiometabolic phenotyping and comparatively crude oral exposure measurement. Recognizing and explicitly addressing these challenges is essential for responsible interpretation of findings and for guiding future methodological improvements.

One of the most pervasive limitations is exposure misclassification, particularly with respect to oral health variables. In many biobanks, oral health is assessed through self-reported symptoms or simplified questionnaire items that lack clinical validation against standardized periodontal examinations. Such measures may capture aspects of oral disease burden but do not reliably distinguish between gingivitis, periodontitis, or other oral conditions, nor do they reflect disease severity or extent. Non-differential misclassification of oral exposures is likely to attenuate true associations with cardiometabolic outcomes, potentially leading to underestimation of effect sizes or inconsistent findings across studies. Differential misclassification may also occur if symptom reporting varies systematically by age, socioeconomic position, health literacy, or comorbidity status, thereby introducing bias that is difficult to quantify.

Residual confounding represents a second major challenge. Oral health and cardiometabolic disease share a wide array of upstream determinants, including smoking, diet, physical activity, psychosocial stress, education, income, and access to healthcare. Although large-scale datasets often include detailed covariate information, measurement error in these variables and unmeasured confounders—such as lifetime dental care utilization, oral hygiene practices, or early-life socioeconomic conditions—may persist. Even sophisticated multivariable adjustment cannot fully eliminate confounding in observational settings, particularly when exposures and outcomes are both influenced by complex, socially patterned processes. Consequently, associations observed in biobank studies should be interpreted as evidence of correlation rather than definitive proof of causation.

Reverse causation and bidirectionality further complicate inference, especially in cross-sectional analyses. As discussed previously, cardiometabolic disease can directly influence oral health through metabolic, inflammatory, and behavioral pathways, making it difficult to determine whether poor oral health precedes or follows cardiometabolic dysfunction. Longitudinal designs mitigate but do not eliminate this concern, as both conditions often develop insidiously over extended periods. Time-varying confounding and feedback loops may obscure temporal ordering, underscoring the need for analytic approaches that explicitly model dynamic relationships rather than assuming unidirectional effects.

Selection bias is another important consideration, particularly in volunteer-based biobanks. Participants in large cohorts such as UK Biobank tend to be healthier, more educated, and more health-conscious than the general population. This “healthy volunteer” effect may limit generalizability and distort exposure–outcome relationships if participation is related to both oral health and cardiometabolic risk. While internal validity may remain acceptable for many analyses, extrapolation to underserved or high-risk populations should be undertaken cautiously. This limitation is especially relevant for oral health research, as oral diseases disproportionately affect socially disadvantaged groups who are often underrepresented in biobank samples.

Beyond internal validity, these selection processes raise important questions regarding the transportability of findings derived from large-scale biobanks. Associations observed in predominantly high-income, healthcare-engaged populations may not directly generalize to settings characterized by different social, behavioral, and healthcare contexts, particularly in low- and middle-income countries where the burden of untreated periodontal disease is high and access to dental care is limited. In such settings, oral health may play a more pronounced role as both a marker of cumulative disadvantage and a contributor to cardiometabolic risk. Explicit consideration of population context, alongside triangulation across cohorts with differing demographic and healthcare profiles, is therefore essential for interpreting oral–cardiometabolic associations and assessing their relevance beyond the source population.

The use of electronic health records and administrative data introduces additional complexities. Diagnostic coding practices for periodontal disease are inconsistent, and absence of a diagnostic code does not reliably indicate absence of disease. Dental claims data may reflect treatment patterns rather than disease prevalence, conflating access to care with underlying pathology. Moreover, medical and dental records are frequently siloed, limiting the ability to construct integrated oral–systemic phenotypes. These issues can lead to both under-ascertainment of oral disease and differential misclassification related to healthcare utilization patterns.

Finally, the increasing availability of high-dimensional data—genomics, metabolomics, imaging—raises concerns regarding multiple testing, overfitting, and interpretability. While such data offer powerful tools for hypothesis generation and causal inference, they require careful analytic planning, transparent reporting, and replication across independent datasets. Failure to address these issues risks generating spurious associations that may further complicate an already complex evidence base.

Collectively, these methodological challenges do not negate the value of large-scale oral–cardiometabolic research but rather define the conditions under which its findings should be interpreted. Progress in this field will depend not only on expanding data resources but also on improving oral health measurement, strengthening integration between dental and medical data systems, and adopting analytic frameworks that explicitly acknowledge uncertainty, bias, and bidirectionality. Narrative reviews that clearly articulate these limitations play a critical role in setting realistic expectations and guiding more rigorous future investigations.

## Future directions: from biobanks to precision oral–cardiometabolic health

The convergence of biobank infrastructure, electronic health records, and advanced analytic approaches presents a pivotal opportunity to move oral–cardiometabolic research beyond descriptive associations toward more integrated and policy-relevant insights. Realizing this potential will depend on coordinated advances in oral health data capture, cross-domain data integration, and analytic rigor.

A central priority is the improvement and harmonization of oral health measurement within large-scale datasets. While self-reported oral health proxies have enabled hypothesis generation at unprecedented scale, their limitations underscore the need for enhanced dental phenotyping. Future biobank initiatives would benefit from standardized oral health modules, structured periodontal diagnosis fields, linkage with dental electronic records, and incorporation of basic treatment histories or tooth loss timelines. Even modest improvements in oral data granularity could substantially strengthen exposure validity and improve interpretability.

Equally important is the integration of medical and dental data systems. Record linkage between dental claims, periodontal treatment records, oral imaging, and existing cardiometabolic datasets would enable more precise characterization of disease trajectories, temporal sequencing, and treatment effects. Such integration is also critical for addressing health inequalities, as it allows investigation of how access to preventive and therapeutic dental care modifies cardiometabolic risk across populations.

Advances in genomics and other omics further expand the analytic landscape of oral–cardiometabolic research. The increasing availability of genetic data within biobanks enables approaches such as Mendelian randomization to help disentangle shared susceptibility from potentially causal effects. Integration of transcriptomic, proteomic, metabolomic, and microbiome data offers additional opportunities to identify intermediate biological signatures that link oral inflammation with metabolic and vascular dysfunction, provided that these analyses are conducted with appropriate methodological rigor and replication.

Artificial intelligence and machine learning methods are likely to play an expanding role in synthesizing high-dimensional data and uncovering complex, non-linear relationships that may not be captured by traditional models. When applied transparently and validated rigorously, such approaches can support risk stratification, interaction detection, and predictive modeling. Importantly, AI-driven analyses should complement, rather than replace, epidemiologic reasoning, with explicit attention to assumptions, bias, and clinical relevance.

From a public health perspective, integrating oral health into cardiometabolic disease surveillance aligns with broader efforts to address non-communicable diseases through precision public health. Large-scale datasets can inform targeted prevention strategies by identifying populations in which poor oral health clusters with cardiometabolic risk due to shared biological, behavioral, or social determinants. Framing oral health as a component of chronic disease prevention, rather than as an isolated outcome, may ultimately support more holistic and equitable approaches to population health.

## Conclusions

The expanding availability of biobanks and large-scale epidemiologic datasets has opened new avenues for investigating the complex relationship between oral health and cardiometabolic disease. While early research was constrained by fragmented data and limited integration of dental variables, contemporary resources now allow population-level analyses that can address longstanding questions regarding association, bidirectionality, and potential mechanisms. This review highlights both the promise and the limitations of these approaches, emphasizing the need for cautious interpretation and methodological transparency.

Evidence to date supports a biologically plausible and epidemiologically consistent association between poor oral health—particularly chronic periodontal inflammation—and adverse cardiometabolic outcomes. These links are underpinned by shared inflammatory pathways, microbial dysbiosis, metabolic dysregulation, and vascular dysfunction, operating within a broader context of genetic susceptibility and social determinants of health. At the same time, cardiometabolic diseases such as diabetes and obesity exert reciprocal effects on oral tissues, reinforcing the concept of bidirectionality rather than a unidirectional causal pathway.

Large-scale datasets excel in capturing cardiometabolic outcomes and enabling longitudinal analyses, but remain limited by imprecise oral health measurement and potential bias. Progress in this field will depend on improved dental data integration, strategic use of complementary datasets, and adoption of analytic frameworks that explicitly acknowledge uncertainty, confounding, and reverse causation. When interpreted alongside mechanistic and clinical evidence, findings from biobank-based studies can meaningfully advance understanding of oral health as a component of cardiometabolic risk.

Harnessing biobanks and population-based data offers a powerful, if still evolving, platform for oral–cardiometabolic research. By bridging dentistry and population health, these resources have the potential to inform more integrated prevention strategies, support precision public health initiatives, and ultimately contribute to reducing the global burden of cardiometabolic disease. Continued investment in data integration, interdisciplinary collaboration, and rigorous methodology will be essential to fully realize this potential.
